# Electroacupuncture Inhibits Autophagy of Neuron Cells in Postherpetic Neuralgia by Increasing the Expression of miR-223-3p

**DOI:** 10.1155/2021/6637693

**Published:** 2021-03-08

**Authors:** Jing Zou, Xueyang Dong, Ke Wang, Jing Shi, Ning Sun

**Affiliations:** ^1^Department of Neurobiology, School of Basic Medicine, Tongji Medical College of Huazhong University of Science and Technology, China; ^2^Department of Acupuncture & Moxibustion, Wuhan Hospital of Integrated Chinese & Western Medicine, Tongji Medical College of Huazhong University of Science and Technology, China; ^3^Wuhan Hospital of Integrated Chinese & Western Medicine, China; ^4^Acupuncture College, Hubei University of Traditional Chinese Medicine, China

## Abstract

Postherpetic neuralgia (PHN) is a complication of herpes zoster viral infection. Its main manifestations are continuous or intermittent burning-like and electroshock-like pain in the affected nerves. Electroacupuncture (EA) is widely used in clinical treatment and exerts effects in alleviating neuropathic pain. In this study, we investigated the effect and underlying mechanism of EA on PHN. Sprague-Dawley rats were treated with resiniferatoxin (RTX) to establish a PHN model and subjected to EA and/or miR-223-3p overexpression (OV) or interference. Mechanical withdrawal latency was measured as an indication of pain sensitivity. Hematoxylin-eosin staining and transmission electron microscopy were performed to observe neuron cell morphology and autophagic vacuoles, respectively. ELISA was performed to detect reactive oxygen species (ROS) production and the levels of tumor necrosis factor- (TNF-) *α*, inducible nitric oxide synthase (iNOS), interleukin- (IL-) 6, and IL-10. Changes in autophagy and apoptosis-related miRNAs were detected by immunofluorescence and qRT-PCR, respectively. In RTX-treated rats, OV and EA reduced pain sensitivity, decreased the number of eosinophils, and increased that of nerve cells. ROS generation and the levels of TNF-*α* and iNOS were significantly reduced, while those of IL-6 and IL-10 were increased. OV and EA induced fewer autophagic vacuoles than those in the model group. The expression of autophagy-related protein microtubule-associated protein 1 light chain 3-II, ATG9, and Rab1 was decreased by OV and EA, whereas that of P62 was increased. qRT-PCR revealed that miR-223-3p expression in the model group decreased but was increased by EA. EA inhibits neuron cell autophagy in PHN by increasing miR-223-3p expression.

## 1. Introduction

Neuralgia is a neurological disease caused by nerve injury or dysfunction and is mainly characterized by abnormal and spontaneous pain and hyperalgesia [1, 2]. Mechanical damage, viral infections, and malignant tumors can cause neuropathic pain. Postherpetic neuralgia (PHN) is a common complication of patients with herpes zoster viral infection. The main manifestations of PHN are continuous or intermittent burning-like and electroshock-like pain in the affected parts of the nerve. Currently, there is no ideal treatment for PHN. An understanding of the pathogenesis of PHN and the development of effective treatment have become urgent problems to be solved.

MicroRNAs (miRNAs) are noncoding RNAs with 22-24 nucleotides that can regulate gene expression after transcription by binding to the 3′-untranslated region of its corresponding messenger RNA [3]. miRNAs participate in the regulation of multiple biological activities in the body and are involved in the modulation of disease progression [4–8]. In recent years, studies have found that miRNAs play important roles in the nervous system and are involved in the occurrence and development of a variety of neurological diseases [9–11]. Xie et al. observed that miR-183 inhibited the occurrence of neuropathic pain by inhibiting the mammalian target of rapamycin/vascular endothelial growth factor signaling [12]. Su et al. found that miR-30b alleviated neuropathic pain [13]. miR-93 and miR-203 have also been shown to be useful in inhibiting the development of neuropathic pain [14]. Therefore, miRNAs may be a potential therapeutic target in the treatment of neuropathic pain.

Acupuncture, which is derived from traditional Chinese medicine, has been used in clinical treatment for thousands of years [15] and exerted effects in alleviating neuropathic pain [16]. In a rat model of neuropathic pain, the expression of TRPV1 was significantly increased, and sensitivity to pain was significantly decreased after electroacupuncture (EA) intervention [17]. Wang et al. applied EA at the Taichong (LR3) acupoint in spontaneously hypertensive rats and subjected the bone marrow of the experimental animals to high-throughput miRNA sequencing [18, 19]. They observed that the expression of multiple miRNAs had been altered to varying degrees, suggesting that acupuncture may exert a therapeutic effect against hypertension by regulating the activity of miRNAs.

Autophagy refers to the process of transporting nonfunctional or damaged proteins, organelles, and macromolecule complexes into lysosomes for degradation and removal to maintain homeostasis of the internal environment. There are three types of autophagy: macrophagy, small autophagy, and chaperone-mediated autophagy. They are important for cell differentiation, survival, and internal environment stability and play an important role in tumors, immune system diseases, and neurodegenerative diseases. The Rab protein family plays a crucial role in the process of autophagy [20]. Rab1, which has two subtypes (Rab1a and Rab1b), participates in the process of autophagy, inhibits the expression of Rab1b by interacting with RFP-LC337, and reduces the production of autophagosomes [21]. Huang revealed that Rab1b is expressed in all autophagosomes with positive microtubule-associated protein 1 light chain 3 (LC3) [22]. Previous studies of our group demonstrated that EA significantly increased the expression of miR-7a-5p, miR-233-5p, and other miRNAs [23]. In previous experiments, we found that the expression of miR-223-3p was significantly decreased after resiniferatoxin (RTX) treatment but increased significantly after EA. We hypothesized that EA affects the autophagy of PHN neuron cells by increasing the expression of miR-223-3p, thereby exerting its therapeutic effect. In this study, we constructed a rat model of neuropathic pain using RTX, and RTX-treated rats were subjected to EA and/or miR-223-3p overexpression/interference. This was achieved by evaluating pain sensitivity, inflammatory factor secretion, and the expression of autophagy-related proteins.

## 2. Materials and Methods

### 2.1. Transfection of Stable miR-223-3p Overexpression/Interference Lentiviral Vectors

miR-223-3p overexpression vector pLVX-IRES-ZsGreen1 (miR223-3p sequence: 5′-TCTGGCCTTCTGCAGTGTTACGCTCCGTGTATTTGACAAGCTGAGTTGGACACTCTGTGTGGTAGAGTGTCAGTTTGTCAAATACCCCAAGTGTGGCTCATGCTTATCAG-3′) and interference vector pSICOR (sequence: 5′-TGTCAGTTTGTGAAATACCCC-3′) were purchased from Addgene (Cambridge, MA, USA). T293 cells were seeded in T25 petri dishes, and when they reached 70–80% confluence, they were transfected with pLVX-IRES-ZsGreen1-miR-223-3p or pSICOR-shmiR-223-3p using Lipofectamine 2000 (Invitrogen, CA, USA) following the manufacturer's instruction. Virus titer was determined by immunofluorescence.

### 2.2. Animals

140 male Sprague-Dawley rats (240–260 g) were purchased from the experimental animal center of the Hubei Academy of Medical Sciences. All rats were kept in individual cages under a 12/12 h light/dark cycle (23–25°C, 40–60% relative humidity) and given free access to food and water during the experiment. Before the formal experiments, the mice were adaptively fed for one week. All experimental procedures in the present study were performed in accordance with the requirements of the Ethics of Animal Experiments and approved by the Animal Care and Use Committee of the Wuhan Myhalic Biotechnology Co. Ltd.

RTX (250 *μ*g/kg, LC Laboratories, USA) was dissolved in a mixture of 10% Tween 80 and 10% ethanol in normal saline and injected in the abdominal cavity of experimental rats according to Wu et al.'s methods [24]. A mixture of 10% Tween 80 and 10% ethanol in normal saline was injected as the control.

Animals were injected with lentiviral vectors three days before the experiment. According to previously reported methods [25, 26], after the rats were anesthetized with 2.5% isoflurane, 10 *μ*l of pLVX-IRES-ZsGreen1-mir-223-3p (miR-223-3p overexpression, OV) or pSICOR-shmiR-223-3p (miR-223-3p interference, IV) vectors was injected into the lumbar vertebrae between L5 and L6 at 1 × 10^9^ TU/ml once a week. Intrathecal injection of empty pLVX-IRES-ZsGreen1 vectors (EV) was also performed. EA was performed one week after RTX injection. The GB30 and GB34 acupoints were stimulated with EA at 2 Hz for 30 min, every other day for 35 days [27]. Sham EA was performed in the same way as EA but without electricity.

### 2.3. Mechanical Withdrawal Latency (MWL)

The MWL was measured according to the method of Sakai et al. [28]. The rats were placed in a transparent glass box of 20 cm × 20 cm before the experiment. After 30 min of adaptation, the rats were placed in a transparent polyethylene pain box with wire mesh (1 cm × 1 cm) at the bottom and allowed to adapt to the environment for 30 minutes. Then, the plantar skin of the rats was stimulated by the von Frey wire pain measurement kit (Aesthesio Danmic Global, USA). The wire was bent and maintained for 6 s, and brisk withdrawal or paw flinching was considered as a positive response. The experiment was performed three times on each rat to obtain the average MWL value, and the data were counted by the “up and down” method [29].

### 2.4. Enzyme-Linked Immunosorbent Assay (ELISA)

Reactive oxygen species (ROS, BIO SWAMP, RA20370) production and the expression of tumor necrosis factor-*α* (TNF-*α*, BIO SWAMP, RA20035), inducible nitric oxide synthase (iNOS, BIO SWAMP, RA20644), interleukin- (IL-) 6 (BIO SWAMP, RA20607), and IL-10 (BIO SWAMP, RA20090) were observed by ELISA. Standard solutions were prepared to generate a calibration curve of concentrations. Samples and enzymes were added to a test tube and incubated at 37°C according to the experimental instructions. The reaction was stopped 10 min after color has appeared and the optical density was measured at 450 nm.

### 2.5. Hematoxylin and Eosin (H&E) Staining

The L2-L6 segment of rat spinal cord tissue was isolated and stripped, washed with double-distilled water, and fixed in 10% neutral formalin for one day. The tissue blocks were placed in an embedding box for dehydration and immersed in wax to be embedded. The tissue samples were cut into sections with a thickness of 4–5 *μ*m. Then, the sections underwent alcohol dehydration, xylene permeabilization, H&E staining, dehydration and permeabilization, and neutral resin sealing. Tissue integrity and inflammatory infiltration in the spinal cord were observed using a microscope.

### 2.6. Transmission Electron Microscopy (TEM)

Rat spinal cord tissues were prefixed in 2.5% glutaraldehyde (10–20 times the tissue volume) at 4°C for 30 min, fixed in 1% osmic acid for 1 h, dehydrated, soaked in a 1 : 1 mixture of acetone and epoxy at 40°C for 6 h, fixed with pure epoxy resin at 40°C for 4 h, and embedded. The samples were then sliced and subjected to double staining and lead citrate staining for 15 min. After rinsing with double-distilled water, the ultrastructure of mitochondria was observed using TEM (HT7700, Hitachi).

### 2.7. Immunofluorescence

Spinal neuron cells were fixed with 4% paraformaldehyde for 10 min at room temperature, permeabilized with 0.3% Triton X-100, and blocked with 5% bovine serum albumin. They were then incubated overnight at 4°C with primary antibodies against NeuN (1 : 200, Abcam, ab177487) and LC3-II (1 : 100, Sanying, 11306-1-AP), followed by incubation with Alexa Fluor 488 secondary antibody (1 : 200, BIO SWAMP, PAB160027) for 30 min at room temperature and counterstaining with DAPI to identify the nuclei. Images were captured with an immunofluorescence microscope (Leica, MD1000).

### 2.8. Quantitative Reverse Transcription Polymerase Chain Reaction (qRT-PCR)

The expressions of miR-133c, miR451-5p, miR-7a-5p, miR135a-5p, miR-486, and miR-223-3p in spinal cord tissues were detected by qRT-PCR. Total RNA was isolated using an RNA extraction kit. Reverse transcription was performed with the following reaction procedure: 42°C, 60 min; 70°C, 15 min; and holding at 16°C. The product of reverse transcription was stored at -20°C.

The prepared cDNA was used in qPCR using primers listed in Table 1. The reaction procedure was 95°C, 3 min for denaturation; 95°C, 5 s and 56°C, 10 s and 72°C, 25 s for 39 cycles; 65°C, 5 s; and 95°C, 50 s. The results were analyzed using the 2^-*ΔΔ*CT^ method.

### 2.9. Western Blot

Proteins were extracted from the L2–L6 lumbar segment of the spinal cord, and their concentration was measured using a bicinchoninic acid protein assay kit (Beyotime, China). Total proteins were separated in 12% sodium dodecyl sulfate-polyacrylamide gel electrophoresis and transferred to polyvinylidene fluoride membranes. The membranes were blocked with 5% milk in Tris-buffered saline (pH 7.6) containing 0.1% Tween 20, incubated with specific primary antibodies overnight at 4°C, and incubated with horseradish peroxidase-conjugated secondary antibody for 2 h at room temperature. The primary antibodies used included anti-LC3-II/I (1 : 1000, 4108, CST), anti-Rab1 (1 : 1000, ab97956, Abcam), anti-autophagy-related protein 9 (ATG9, 1 : 1000, ab108338, Abcam), anti-P62 (1 : 2000, ab155686, Abcam), and anti-GAPDH (1 : 5000, 10494-1-AP, Proteintech). After three washes with PBS/Tween 20, the membranes were incubated with horseradish peroxidase-conjugated secondary goat anti-rabbit IgG (1 : 20000, PAB160011, BIO SWAMP). Protein bands were visualized by enhanced chemiluminescence color detection (Tanon-5200, TANON) and analyzed using AlphaEase FC gel image analysis software.

### 2.10. Luciferase Reporter Assay

The Rab1 3′-UTR was designed and synthesized by Nanjing Kingsray Bio (Nanjing, China). The target sites of the human Rab1 3′-UTR segments for miR-223-3p were amplified by PCR. The PCR product was inserted into the luciferase reporter pGLO dual luciferase reporter vector (Thermo Fisher Scientific, Waltham, MA, USA) to generate the Rab1 3′-UTR wild type (WT). The Rab1 3′-UTR mutant (MUT) was prepared by mutating the seed regions of the miR-223-3p binding sites using a site-directed mutagenesis kit (Takara Bio, Shiga, Japan). For reporter assays, 2 × 10^5^ cells were seeded in 96-well plates and cotransfected with 0.2 *μ*g of Rab1 3′-UTR or mutant Rab1 3′-UTR and 0.5 *μ*l miR-223-3p mimics or inhibitor using Lipofectamine 2000. Each transfection was performed in triplicate, and luciferase activity was detected by the Dual-Luciferase Reporter Assay System (Promega, Madison, WI, USA) after transfection for 48 h.

### 2.11. Statistical Analysis

Data are expressed as the mean ± standard deviation. To analyze the differences between groups, data comparison was performed by *t* tests and one-way analysis of variance using SPSS 22 statistical software. *P* < 0.05 was considered statistically significant.

## 3. Results

### 3.1. Effect of EA on RTX-Induced Mechanical Allodynia

**Figure 1:**
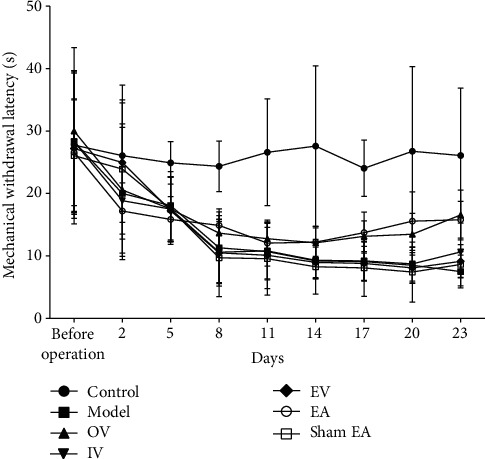
Effect of EA on RTX-induced mechanical allodynia. *n* = 20. Time course of mechanical withdrawal threshold in response to von Frey filaments. Control: Sprague-Dawley rats; model: injected with RTX; OV: 10 *μ*l of pLVX-IRES-ZsGreen1-mir-223-3p vectors was injected into the lumbar vertebrae between L5 and L6 at 1 × 10^9^ TU/ml once a week; IV: 10 *μ*l of pSICOR-shmiR-223-3p vectors was injected into the lumbar vertebrae between L5 and L6 at 1 × 10^9^ TU/ml once a week; EV: 10 *μ*l of empty pLVX-IRES-ZsGreen1 vectors was injected into the lumbar vertebrae between L5 and L6 at 1 × 10^9^ TU/ml once a week; and EA: the GB30 and GB34 acupoints were stimulated with EA at 2 Hz for 30 min. Sham EA: performs in the same way as EA but without electricity. EA and miR-223-3p were applied every other day starting from seven days after RTX injection.

To detect the effect of EA and miR-223-3p on mechanical pain threshold, we evaluated the tactile sensitivity of rats after RTX administration. The baseline threshold was similar in all groups before RTX intervention, as demonstrated in Figure 1. EA and miR-223-3p were applied every other day starting from seven days after RTX injection. After eight days of EA and miR-223-3p treatment, the mechanical threshold began to increase and gradually increased until 23 days after EA treatment (Figure 1).

### 3.2. Overexpression of miR-223-3p and EA Treatment Inhibited Neuronal Injury and Apoptosis

**Figure 2:**
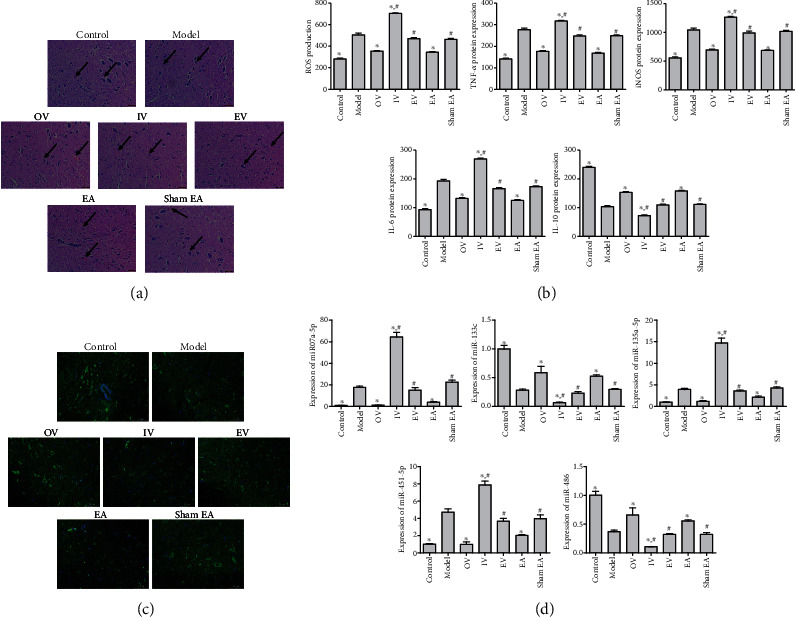
Effect of miR-223-3p overexpression and EA on neuronal injury and apoptosis. (a) H&E staining of spinal cord tissue (scale bar = 50 *μ*m). (b) ROS production and protein expression of TNF-*α*, iNOS, IL-6, and IL-10 were measured by ELISA in serum. (c) Immunofluorescence of nerve cells (scale bar = 50 *μ*m). (d) qRT-PCR of the expression of miR-7a-5p, miR-133c, miR-451-5p, miR-486, and miR-135a-5p in spinal cord tissues. The results are presented as the mean ± SD, *n* = 3. ^∗^*P* < 0.05 vs. model, ^#^*P* < 0.05 vs. OV.

To investigate the effect of miR-223-3p and EA on the structure and apoptosis of nerve cells, rats were euthanized (200 mg/kg pentobarbital, intraperitoneal injection) after 35 days of RTX treatment. Blood and the L2-L6 segments of the spinal cord nerve tissue were collected for detection. Compared with the control group, RTX-treated rats showed an increase in the number of eosinophils (Figure 2(a)). After OV and EA treatment, the number of eosinophils decreased. A large amount of eosinophil infiltration was observed in the IV group, suggesting that miR-223-3p and EA may have anti-inflammatory and inhibitory effects on nerve cell injury. To verify this, ROS production and the expression of TNF-*α*, iNOS, IL-6, and IL-10 in the serum were detected by ELISA (Figure 2(b)). Compared with the model group, ROS production and the expression of TNF-*α*, iNOS, and IL-6 in the OV and EA groups were significantly lower (*P* < 0.05), and the expression of IL-10 was significantly higher. The opposite trend was observed in the IV group (*P* < 0.05), and there was no significant difference between OV and EA (*P* > 0.05). As shown in Figure 2(c), there were fewer nerve cells in the model group than in the control group. Compared with the model group, the number of nerve cells was increased in the OV and EA groups, while that in the IV group decreased. We detected the changes in apoptosis-related miRNAs in the spinal cord by qRT-PCR to explore whether miR-223-3p and EA have protective effects on nerve cells. As shown in Figure 2(d), compared with the control, the expression of miR-133c and miR-486 decreased in the model group (*P* < 0.05), while that of miR-451-5p, miR-135a-5p, and miR-7a-5p increased (*P* < 0.05). Compared with the model group, the expression of miR-451-5p, miR-135a-5p, and miR-7a-5p increased in the IV group (*P* < 0.05) and that of miR-133c and miR-486 decreased (*P* < 0.05), while in the other groups, the expression of miR-451-5p, miR-135a-5p, and miR-7a-5p decreased (*P* < 0.05) and that of miR-133c and miR-486 increased (*P* < 0.05). The findings suggest that miR-223-3p and EA could inhibit neuronal apoptosis.

### 3.3. Overexpression of miR-223-3p and EA Treatment Inhibited Autophagy in Neuron Cells

**Figure 3:**
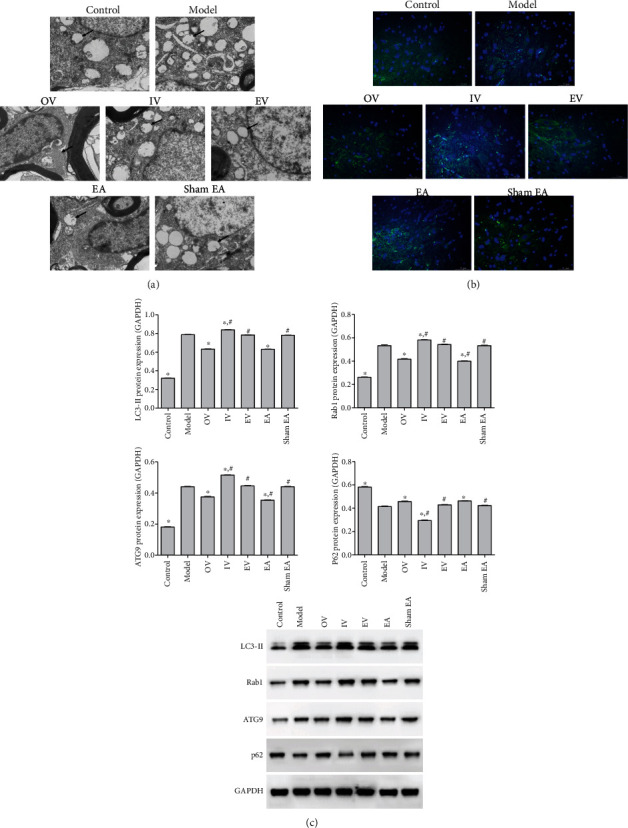
Effect of miR-223-3p overexpression and EA on neuronal autophagy. (a) Detection of autophagic vacuoles in spinal cord neurons by transmission electron microscopy (scale bar = 2 *μ*m). (b) Immunofluorescence of the protein expression of LC3-II (scale bar = 50 *μ*m). (c) Protein expression of LC3-II, Rab1, ATG9, and P62 was measured by western blot. The results are presented as the mean ± SD, *n* = 3. ^∗^*P* < 0.05 vs. model, ^#^*P* < 0.05 vs. OV.

The formation of autophagic vacuoles in spinal cord neurons was observed using TEM. There were more autophagic vacuoles in the model group than in the control, OV, and EA groups (Figure 3(a)). Compared with the control group, the LC3-II content in the model group increased. The LC3-II expression in the IV group increased but decreased in the EA and OV groups compared with that in the model group (Figure 3(b)). To further clarify the effect of miR-223-3p and EA on nerve cell autophagy, the expression of autophagy-related proteins was evaluated (Figure 3(c)). Compared with the model group, the expression of LC3-II, ATG9, and Rab1 in the OV and EA groups decreased significantly (*P* < 0.05) and that of P62 increased significantly, and IV showed the opposite trend (*P* < 0.05). These results revealed that miR-223-3p and EA inhibited nerve cell autophagy.

### 3.4. Rab1 Was a Target of miR-223-3p in Glial Cells

**Figure 4:**
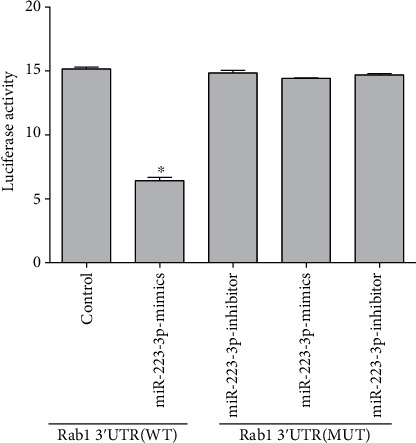
Rab1 was a target of miR-223-3p in nerve cells. The results are presented as the mean ± SD, *n* = 3. ^∗^*P* < 0.05 vs. control.

As the result shows in Figure 4, luciferase activity in the group cotransfected with miR-223-3p mimics and Rab1 3′-UTR wild type was significantly decreased (*P* < 0.05) compared to that in the other group, suggesting that Rab1 was the target of miR-223-3p.

### 3.5. EA Promoted the Expression of miR-223-3p

**Figure 5:**
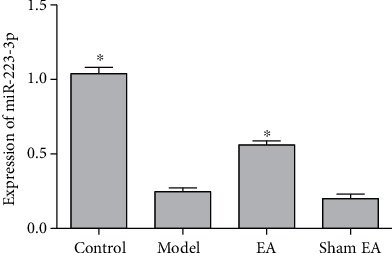
Effect of EA on miR-223-3p expression. The results are presented as the mean ± SD, *n* = 3. ^∗^*P* < 0.05 vs. model.

To study whether EA exerts its therapeutic effect by affecting the expression of miR-223-3p, we used qRT-PCR to detect the expression of miR-223-3p (Figure 5). Compared with the control group, the expression of miR-223-3p in the model group decreased significantly (*P* < 0.05) but was significantly increased by EA (*P* > 0.05). Sham EA did not have an effect on miR-223-3p expression.

## 4. Discussion

PHN is a persistent neuropathic pain that causes severe social and economic consequences. Acupuncture, a common treatment in traditional Chinese medicine, has been used to relieve pain for centuries. In particular, EA is a type of treatment involving the electric stimulation of specific points of the human body. In this study, we observed the effect of EA on neuron cell autophagy by constructing an in vivo neuropathic pain model.

Wu et al. showed that injection of RTX reduced the thermal sensitivity of rats and led to persistent tactile abnormalities, similar to the symptoms of clinical PHN patients [27]. Therefore, we used RTX to establish a rat model in this study and found that RTX injection increased the tactile sensitivity. The analgesic effect of EA has been confirmed by many clinical observations and experimental studies [30, 31]. In particular, EA relieved pain by regulating the dynorphin/kappa opioid receptor system [32] and alleviated neuropathic pain by inhibiting the secretion of prostaglandin E2 [33]. In this study, we found that when the GB30 and GB34 acupoints were stimulated by EA at 2 Hz, the tactile sensitivity of rats was significantly reduced. This is the same result as that shown by Wu et al. [27].

Our previous research showed that miR-223-3p might be a key miRNA in the relief of neuralgia by EA [23]. miR-223-3p was reported to suppress the activity of inflammasomes and was involved in regulating inflammation [34, 35]. Under physiological conditions, pain signals are transmitted from the peripheral nerve to the spinal cord via A*δ* and C fibers. This causes the release of excitatory amino acids and substance P, thereby depolarizing the pain-transmitting neurons to produce action potentials that are transmitted to the brain center to produce the sensation of pain. During this process, glial cells are in a resting state. However, with the continuous introduction of pain signals in the pathological state, N-methyl-D-aspartate receptors on pain neuron membranes will also be activated, resulting in increased intracellular Ca^2+^ concentration and nitric oxide synthesis, which activate glial cells. Activated glial cells release a large number of neuroactive substances and inflammatory factors, such as ROS, NO, IL-10, IL-6, and TNF-*α*. These substances act on neurons in the spinal synapses and enhance their sensitivity and responsiveness. The inflammatory cytokines TNF-*α* and IL-6 interact with sodium-calcium channels on the cell membrane, rapidly increasing the excitability of neurons and leading to pain. Inflammatory factors such as TNF-*α* and IL-6 not only promote the release of traditional pain-causing substances but also enhance their release through autocrine and paracrine means in synergy with each other, causing persistent pain [36]. Our results showed that the infiltration of inflammatory cells in the spinal cord was decreased in the OV and EA groups. We further revealed that OV and EA significantly inhibited ROS production and the expression of proinflammatory factors TNF-*α*, iNOS, and IL-6 and increased the activity of the anti-inflammatory factor IL-10. IL-6 and TNF-*α* are inflammatory factors that are most closely associated with the acceleration of pain [37]. IL-10 is highly active and, in addition to inhibiting proinflammatory cytokines, promotes the production of other anti-inflammatory factors and blocks the cytokine cascade reaction to play an analgesic role [38]. Our results suggest that EA and OV inhibited the expression of proinflammatory factors and promoted the activity of anti-inflammatory factors to relieve neuropathic pain. Sekiguchi et al. revealed that various types of neuropathic pain are associated with apoptosis of dorsal root ganglia (DRG) and spinal cord neurons [39], wherein inhibition of neuronal apoptosis promotes the transmission of nociceptive information. This results in the increased excitation of nociceptive neurons transmitted from the DRG to the spinal cord, leading to hyperalgesia [40]. In previous studies, we found that miR-133c, miR-451-5p, miR-486, miR-135a-5p, and miR-7a-5p may be key factors involved in the effect of EA in alleviating neuropathic pain. Xu et al. observed that the increased expression of miR-133 inhibited cardiomyocyte apoptosis [41]. Sun et al. found that upregulation of miR-486 expression in cardiomyocytes effectively reduced the activation of the Bcl-2-related mitochondrial apoptotic pathway, thereby protecting cardiomyocytes from apoptosis [42]. Liu et al. observed that miR-451, a tumor suppressor, selectively increased the sensitivity of ERCC1-high non-small cell lung cancer cells to cisplatin [43]. miR-135-5p inhibited adipocyte formation through the Wnt/beta-catenin pathway [44], and miR-7a-5p inhibited melanoma cell migration and invasion [45]. In this work, OV and EA increased the expression of miR-133c and miR-486 while decreasing that of miR-451-5p, miR-135a-5p, and miR-7a-5p. These results suggested that EA and miR-223-3p reduced neuronal damage and inhibited cell apoptosis.

LC3 is the first mammalian protein that has been confirmed to bind specifically to autophages. Its two forms, LC3-I and LC3-II, are essential in the formation and extension of autophages. During autophagy, LC-I dispersed in the cytoplasm is coupled with phosphatidylethanolamine on the surface of the autophagic membrane to form LC3-II, which is regarded as the most important marker of autophagy. LC3-II participates in the formation of the autophagic membrane, combines with lysosomes, and is degraded under the catalysis of lysosomal enzymes.

A total of 38 autophagy-related (ATG) genes have been identified in eukaryotic cells, among which mATG9 is the only transmembrane protein. Its transport is regulated by TBC1D14 and TRAPPIII complexes, the recruitment of which can activate Rab1 to promote Golgi transport and recover mATG9 to maintain autophagic flux [46]. Chen et al. found that the increase in the autophagic activity of glial cells was directly proportional to the increase in inflammation and the decrease in pain threshold in rats [47]. After intrathecal injection of 3-methyladenine, the pain threshold of rats was significantly increased and the inflammation response was decreased, suggesting that inhibition of glial cell autophagy may reduce symptoms of neuropathic pain. In this study, OV and EA increased the pain threshold, decreased inflammatory response, reduced autophagic vesicles, downregulated the expression of autophagy-related proteins, and upregulated that of P62 in rats, in accordance with previous studies. It is suggested that overexpression of miR-223-3p and EA treatment could alleviate neuropathic pain by inhibiting autophagy of neuron cells. We further examined the relationship between EA and miR-223-3p and showed that EA induced the expression of miR-223-3p, suggesting that the inhibition of neuron cell autophagy by EA may be achieved by regulating miR-223-3p.

In conclusion, our study demonstrated that EA reduced neuron cell apoptosis and inflammation and increased the mechanical pain threshold of rats with PHN. EA may inhibit autophagy of neuron cells by increasing the expression of miR-223-3p; the mechanism may be related to the targeting regulation of Rab1 by miR-223-3p.

## Figures and Tables

**Table 1:** Primer sequences.

Primer	ssSequence (5′-3′)
miR223-RT	CTCAACTGGTGTCGTGGAGTCGGCAATTCAGTTGAGGGGGTATT
miR223-F	GGGTGTCAGTTTGTCA
miR133c-RT	CTCAACTGGTGTCGTGGAGTCGGCAATTCAGTTGAGTTGGTCCC
miR133c-F	GGGCAGCTGGTTGAAG
miR451-RT	CTCAACTGGTGTCGTGGAGTCGGCAATTCAGTTGAGAACTCAGT
miR451-F	GGGAAACCGTTACCATT
miR486-RT	CTCAACTGGTGTCGTGGAGTCGGCAATTCAGTTGAGCTCGGGGC
miR486-F	GGGTCCTGTACTGAGCT
miR135a-RT	CTCAACTGGTGTCGTGGAGTCGGCAATTCAGTTGAGTCACATAG
miR135a-F	GGGTATGGCTTTTTATTC
miR7a-RT	CTCAACTGGTGTCGTGGAGTCGGCAATTCAGTTGAGACAACAAA
miR7a-F	GGGTGGAAGACTAGTGAT
U6-F	CTCGCTTCGGCAGCACA
U6-R	AACGCTTCACGAATTTGCGT

## Data Availability

The data sets generated for this study are available on request to the corresponding author.
